# Genome-Wide Association Analysis Identified Quantitative Trait Loci (QTLs) Underlying Drought-Related Traits in Cultivated Peanut (*Arachis hypogaea* L.)

**DOI:** 10.3390/genes15070868

**Published:** 2024-07-02

**Authors:** Phat Dang, Jinesh Patel, Ron Sorensen, Marshall Lamb, Charles Y. Chen

**Affiliations:** 1 USDA-ARS, National Peanut Research Laboratory, Dawson, GA 39842, USA; ron.sorensen@usda.gov (R.S.); marshall.lamb@usda.gov (M.L.); 2 Department of Crop, Soil and Environmental Sciences, Auburn University, Auburn, AL 36849, USA; jdp0078@auburn.edu (J.P.); cyc0002@auburn.edu (C.Y.C.)

**Keywords:** diversity panel, GWAS, drought, peanut

## Abstract

Drought is a destructive abiotic stress that affects all critical stages of peanut growth such as emergence, flowering, pegging, and pod filling. The development of a drought-tolerant variety is a sustainable strategy for long-term peanut production. The U.S. mini-core peanut germplasm collection was evaluated for drought tolerance to the middle-season drought treatment phenotyping for pod weight, pod count, relative water content (RWC), specific leaf area (SLA), leaf dry matter content (LDMC), and drought rating. A genome-wide association study (GWAS) was performed to identify minor and major QTLs. A total of 144 QTLs were identified, including 18 significant QTLs in proximity to 317 candidate genes. Ten significant QTLs on linkage groups (LGs) A03, A05, A06, A07, A08, B04, B05, B06, B09, and B10 were associated with pod weight and pod count. RWC stages 1 and 2 were correlated with pod weight, pod count, and drought rating. Six significant QTLs on LGs A04, A07, B03, and B04 were associated with RWC stages 1 and 2. Drought rating was negatively correlated with pod yield and pod count and was associated with a significant QTL on LG A06. Many QTLs identified in this research are novel for the evaluated traits, with verification that the pod weight shared a significant QTL on chromosome B06 identified in other research. Identified SNP markers and the associated candidate genes provide a resource for molecular marker development. Verification of candidate genes surrounding significant QTLs will facilitate the application of marker-assisted peanut breeding for drought tolerance.

## 1. Introduction

Peanut (*Arachis hypogaea* L.) is an important oil seed and protein crop that is grown in the United States and around the world, providing high-quality oil and for human nutrition [1]. Total world production is approximately 50 million metric tons, with the United States as fourth behind China, India, and Nigeria [2]. Peanut is grown in many semi-arid areas often with the combination of drought and high temperatures that can significantly reduce yield and seed quality. Even with irrigation, limited water availability during the critical developmental stage can limit plant productivity. The development of drought-tolerant peanut varieties is a sustainable strategy with the potential to be grown under reduced irrigation levels or only under rainfed conditions.

Breeding for drought tolerance has been challenging due to a high environmental component and multiple allelic interactions [3]. Water use efficiency (WUE), the amount of carbon assimilated as plant biomass (or yield) per unit of water used by the crop, has been intensely studied to select drought-tolerant plants. At the leaf level, transpiration efficiency (TE) was defined as biomass per water unit transpired and was considered an intrinsic value of WUE. Surrogate measurements for TE, such as specific leaf area (SLA) and SPAD chlorophyll meter readings (SCMR), have been utilized to select peanut plants under drought [4,5]. In terms of water relations and plant response, measurements of relative water content (RWC) and SLA are important indicators of drought tolerance. Maintaining relatively higher RWC levels among different peanut genotypes and reducing the SLA to reduce water loss may be indicators of drought tolerance [6]. Leaf dry matter content (LDMC) represents the leaf total dry matter that can act as osmolytes to minimize water loss under drought. Yield quality traits such as pod weight and pod count are indicators of good plant performance in different growing environments. Studies have conducted to identify QTLs for seed weight and seed size [7,8]. Yang et al. [9] identified major QTLs on A08 and B06 for peanut yield.

The U.S. peanut germplasm collection is a good resource to identify candidate peanut lines with good quality traits such as drought tolerance and desirable seed quality characteristics for introgression into cultivated peanuts. This resource has been evaluated to identify seed and pod quality traits applying genome-wide association study (GWAS) strategy [10,11] and the identification of ionomic variations [12]. In this study, the U.S. peanut mini-core collection was evaluated for middle-season drought tolerance utilizing environmentally controlled shelters. The phenotyping of physiological traits such as RWC, SLA, and LDMC, yield traits such as pod weight and pod count, and drought rating were performed. Whole-genome scans of high-quality SNPs were associated with phenotyping data to identify significant genomic regions.

## 2. Materials and Methods

### 2.1. Plant Materials and Experimental Treatments

A total of 124 peanut genotypes were evaluated which included 104 plant introductions (PIs) from the U.S. peanut mini-core germplasm collection (USDA, Griffin, GA, USA) [13] and 20 additional PIs selected from the complete US germplasm collection (Supplementary Table S1). This panel encompasses a wide range of plant characteristics from all six peanut botanical peanut varieties: *aequatoriana*, *fastigiata*, *hirsuta*, *hypogaea*, *peruviana*, and *vulgaris*, collected from different locations around the world. Peanuts were planted on 18 May 2017 and 16 May 2018 in a randomized complete block design with 2 replications per genotype. Seeds were planted at the seeding rate of 20 seeds/m in 0.9 m rows, with 0.45 m within row spacing and 0.9 m between rows spacing, in six environmentally controlled rainout shelters (5.5 m × 12.2 m) [14] equipped with metal covers which automatically close as water droplets are detected by a rain detector (Agrowtek, Brookfield, WI, USA). Each shelter was equipped with a set of Water Mark soil moisture sensors (Meter, Pullman, WA, USA) placed in the middle of the plots at the depths of 10 and 20 cm. Prior to and after drought treatment, supplementation irrigation was triggered based on the mean of two soil moisture sensors average below −60 kPa and a total of 12.7 mm of water was applied each time. Fungicide application conformed to UGA best management practices, insecticide was applied as needed, and weeds were manually controlled.

### 2.2. Phenotypic Data Collection

Drought was initiated at 71 days after planting (DAP) with a suspension of supplemental irrigation by covering shelters when rain was detected. Plant leaf sample collections and physiological measurements were performed in 2017 and 2018: (stage 1, 71 DAP; stage 2, 85 DAP; stage 3, 92 DAP; stage 4, 99 DAP) and 7 days after re-irrigation (stage 5, 113 DAP). For measurements of relative water content (RWC), specific leaf area (SLA), and leaf dry matter content (LDMC), a second-nodal tetrafoliate leaf from 3 independent mainstems were collected in the morning (9 to 11 am), put into plastic bags, placed on ice until all samples were collected, and processed the same day. Fresh leaves were weighed then placed completely into large Petri dishes (60 × 15 mm) filled with deionized water and placed under white light lamp for 2 to 3 h to ensure leaves were fully turgid. Leaves were blotted to remove excess water and weighed. Leaf area was determined using the LI-3100 area meter (Li-Cor Biosciences, Lincoln, NE, USA). Relative water content (RWC) was calculated based on the formula: RWC (%) = ((FW − DW)/(TW − DW)) × 100, where FW was fresh weight, TW was turgid weight, and DW was dry weight [15]. SLA was the ratio of leaf area to leaf dry mass and LDMC was the ratio of leaf dry mass to saturated fresh mass. Drought rating was measured based on visual observation that assigns a number ranging from 1 (canopies that have no or little drought symptoms) to 5 (plants with all dried leaves, discolored, and even dead) at the last day of drought treatment during the hottest part of the day (2–4 pm). At the end of the growing season, peanuts were manually harvested at 131 DAP in 2017 and at 141 DAP in 2018, followed by plant/pod separation using a plot thresher (Kangaroy Engineering Works, Kingaroy, Australia). After harvest, inshell peanuts were cured by applying heated air (not to exceed 35 °C) for 2 days, followed by ambient air only for an additional 2 days (if needed) to attain a 9% seed moisture. Total pod count and weight were documented after removing foreign material (plant debris, and rocks, etc.) from each sample.

### 2.3. SNP Genotyping Utilizing SNP Gene Array

To perform SNP genotyping, leaf tissue samples were collected from each accession, and DNA was isolated using the CTAB protocol. The quality and quantity of DNA were assessed using NanoDrop2000 (Thermo Scientific, Waltham, MA, USA). GeneSeek (Neogen, Lincoln, NE, USA) genotyped all the samples on the Axiom_Arachis array [16]. A total of 17,223 SNPs were found to be polymorphic in the association panel.

### 2.4. Statistical Data Analysis

The PROC CORR command of SAS software (SAS Institute Inc., SAS^®^9.2, Cary, NC, USA) was used to analyze correlation between drought rating, pod dry weight, pod count, RWC (stages 1 and 2), SLA (stages 1 and 2), and LDMC (stages 1 and 2) using R program. A correlation matrix was then created using CORRPLOT in R to visually display the correlations. The variance components were estimated using the restricted maximum likelihood (REML) method, and the univariate variance analyses were performed using the standard GLM method in SPSS Statistics Version 24 software (IBM Corp, NY, USA). Broad-sense heritability for each trait was calculated using *H*^2^ = *σ*^2^*_g_*/(*σ*^2^*_g_* + *σ*^2^*_g×e_*/*n* + *σ*^2^*_error_*/*nr*) where *σ*^2^*_g_* was genotypic variance, *σ*^2^*_g×e_* represents variance due to the interaction between genotype and environment, *σ*^2^*_error_* corresponds to the residual (error) variance. Additionally, *n* denotes number of the environments (2 years), and *r* was number of replications (3 in our case) in each environmental trial [17]. Best linear unbiased prediction (BLUP) for the above-mentioned traits for each genotype was calculated using ‘lme4’ package in R4.3.1 software “www.r-project.org (accessed 11 October 2023)” [18]. These values were used to conduct GWAS analysis.

### 2.5. Filtered Markers for LD Decay and Population Structure

**Figure 1:**
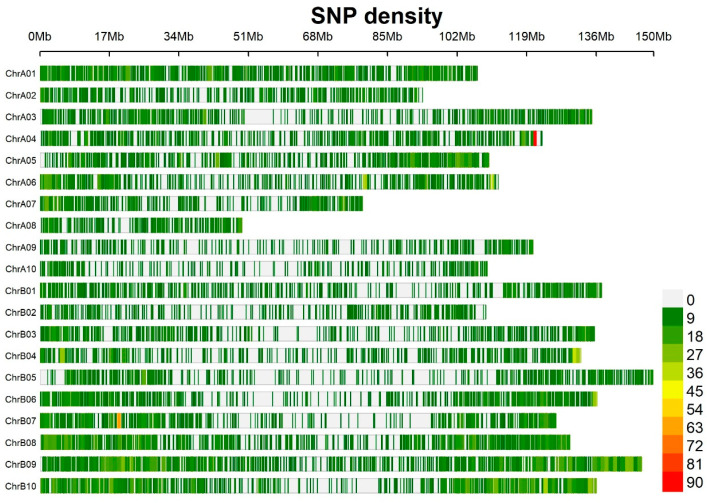
Distribution of 13,382 single nucleotide polymorphisms (SNPs) on 20 chromosomes from the genotyping of the association panel on the peanut genome. Color pattern on each chromosome represents the density of SNP markers in the regions of 1 Mb window size. The x axis represents the physical distance along each chromosome.

The 17,233 SNPs with minor allele frequency (MAF) < 5% or with a missing rate larger than 10% were filtered using Tassel program [19]. After this step, a total of 13,382 SNPs were selected that were distributed on 20 chromosomes (Figure 1) and used to conduct further analysis. Population structure analysis and LD decay were conducted in previous publications [10] using software STRUCTURE v.2.3.4 [20] and PopLDDecay [21], respectively. For structure analysis, the SNP data were used with the admixture model with five iterations of 50,000 burn-in and 50,000 Monte Carlo Markov Chain (MCMC) replications for k = 1 to k = 10. The ideal level of subpopulation was determined by calculating DeltaK using the Structure Harvester [22].

### 2.6. Genome-Wide Association Mapping Analysis

The filter set of markers was used to conduct genome-wide association (GWA) analysis with FarmCPU (fixed and random model circulating probability unification), in GAPIT version 3 package of R [23]. FarmCPU effectively controls false positives and false negatives compared to the generalized linear model (GLM) and the mixed linear model (MLM). The threshold *p* value for genome-wide significance was calculated to be *p* = 9.77 × 10^−4^ or −log10^(*p* value)^ = 3.01 indicating a suggestive association and *p* = 4.8 × 10^−5^ and −log10^(*p* value)^ = 4.31, which indicates a significant association These values were determined using the Bonferroni correction with the estimated number of independent SNPs and LD blocks [10].

### 2.7. Candidate Genes Search

Genome sequence of tetraploid peanut cultivar (*A. hypogaea*, Tifrunner) genome to identify candidate genes [24]. A window of 160 kb was selected based on LD decay on both sides of significantly associated markers. Information related to gene position and annotation was downloaded for *A. hypogaea* v1.0 gene model “https://peanutbase.org (accessed 8 September 2023)” extracted using Python v3.11 script.

## 3. Results

### 3.1. Correlation of RWC, SLA, LDMC, Drought Rating, Pod Count, and Pod Weight

**Figure 2:**
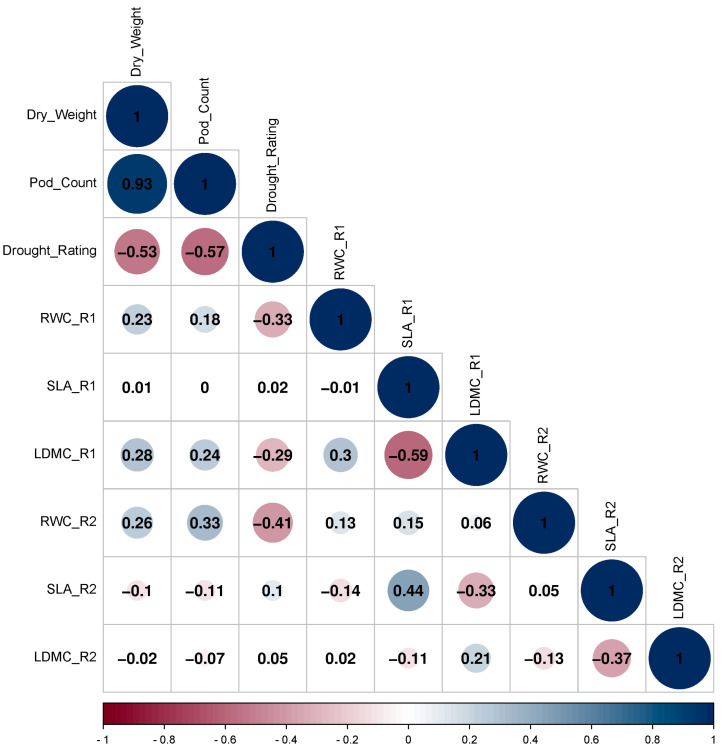
Correlation matrix of pod count, pod dry weight, drought rating, relative water content (RWC), specific leaf area (SLA), and leaf dry matter content (LDMC) (stages 1 and 2) for 2 years average result of both 2017 and 2018. The size and number of the circle indicate the strength of the relationship and correlation coefficient (r). Blue color indicates a positive relationship and red indicates a negative relationship between traits.

Physiological traits such as RWC, SLA, and LDMC were correlated to pod dry weight, pod count, and drought rating. For 2-year average results, both RWC stages 1 and 2 were positively correlated with pod weight and pod count while it was negatively correlated with drought rating (0.23, 0.18, −0.33 and 0.26, 0.33, −0.41) (Figure 2).

SLA stage 1 was positively correlated with SLA stage 2 (0.44) and negatively correlated LDMC stage 1 (−0.59). LDMC stage 1 was positively correlated with pod dry weight and pod count while negatively correlated with drought rating (0.28, 0.24, −0.29). SLA stages 1 and 2, and LDMC stage 2 have no significant correlations with pod weight, pod count, and drought rating. Comparing 2017 to 2018, pod weight and pod count for 2018 were reduced approximately by half (0.56, 0.52) (Supplementary Figure S1). Drought rating for 2018 was positively correlated with drought rating for 2017 (0.19). Drought rating for 2017 was negatively correlated with dry weight and pod count for 2017 (−0.53, −0.56) and drought rating for 2018 was negatively correlated with dry weight and pod count for 2018 (−0.44, −0.45). Broad-sense heritability estimates for pod dry weight, pod count, and drought rating were 69.75, 66.31, and 28.89, respectively (Table 1). For RWC, SLA, and LDMC stages 1 and 2, the heritability estimates were 14.11, 40.17, 41.18 and 37.71, 35.18, 0, respectively.

### 3.2. Distribution of SNP Markers, Linkage Disequilibrium, and Population Structure

A total of 124 peanut genotypes were genotyped utilizing a peanut Axium_Arachis gene array. A total of 17,223 SNPs were identified to be polymorphic. After the selection of SNPs with minor allele frequency (MAF) < 5% and filtering for a rate larger than 10%, a total of 13,382 high-quality SNPs were selected that were distributed on 20 chromosomes (Figure 1). Population structure analysis and LD decay were described in a previous publication [10].

### 3.3. Genomic Regions Associated with Quantitative Traits and Search for Candidate Genes

**Figure 3:**
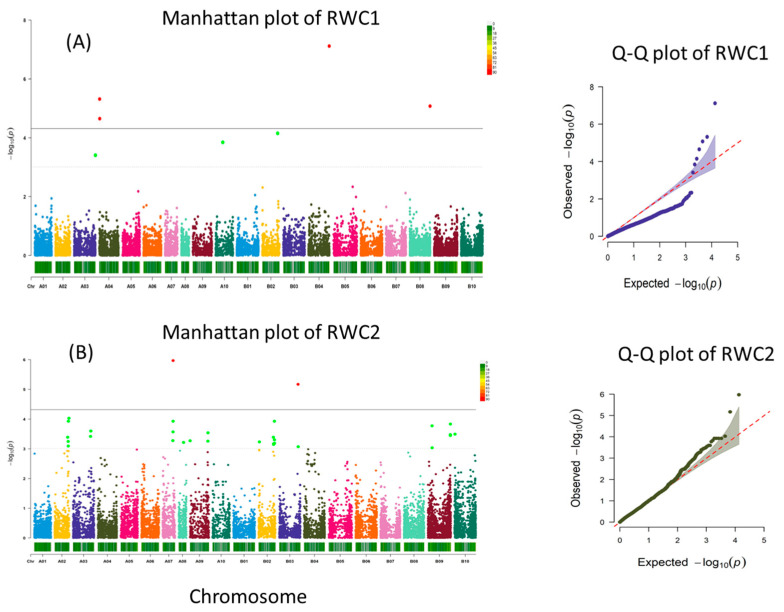

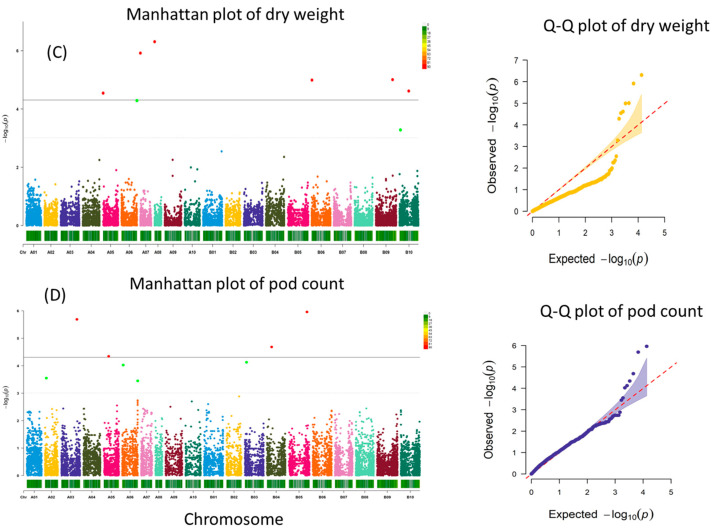
Manhattan and Q–Q plots for RWC stage 1 (**A**), RWC stage 2 (**B**), dry weight (**C**), and pod count (**D**). Significant threshold is marked by solid grey line, and suggestive threshold is marked by a light dotted line in the Manhattan plot.

A total of four, two, one, six, four, and one SNPs were significantly associated with RWC1, RWC2, SLA1, dry weight, pod count, and drought rating, respectively (Figure 3).

#### 3.3.1. Relative Water Content Stage 1

RWC stage 1 identified three suggestive SNP markers on chromosomes A03 (1), A10 (1), B02 (1) and four significant SNPs (Supplementary Table S2). Two significant SNPs (*p* < 4.80 × 10^−6^ and *p* < 2.24 × 10^−5^) were located on chromosome A04 (AX-147219057 and AX-147219130) at positions 2934143 and 3582051, respectively. For marker AX-147219057, the nearby search identified 42 candidate genes (Supplementary Table S3), including lactoylglutathione lyase (*Arahy.5S2G3K*, *Arahy.VPW5NJ*), signal transduction proteins such as serine/threonine-protein phosphatase (*Arahy.SK12UP*, *Arahy.3ML67S*), phosphomethylpyrimidine kinase and protein kinase (*Arahy.DFQ1AC*, *Arahy.P47HRA*), ATP phosphoribosyl transferase (*Arahy.3Q8GGF*), and ATP phosphoribosyl transferase (*Arahy.3Q8GGF*). For marker AX-147219130, the search for genes within proximity identified 27 candidate genes, including squamosa promoter binding protein-like (*Arahy.M2TLIU*), sugar transporter (*Arahy.5QB4T4*), signaling-related proteins such as ankyrin repeat-containing protein (*Arahy.1CD59K*), histidine-phosphotransferase protein (*Arahy.2E9LMX*), protein modification such as endonuclease/exonuclease/phosphatase (*Arahy.S9FSJL*), and protein phosphatase 2C (*Arahy.I5K3IU*). A third highly significant SNP (*p* < 7.60 × 10^−8^) was located on chromosome B04 (AX-147248780) at positions around 130738535 with 20 candidate genes within proximity, including several disease resistance proteins (TIR-NBS-LRR) (*Arahy.RGV79Z*, *Arahy.27BWBV*, *Arahy.QZS0X1*, *Arahy.IB6E9K*, *Arahy.IB6E9K*, *Arahy.27QVSF*, *Arahy.EG26MQ*, *Arahy.Q1WEA7*, *Arahy.59G75N*, *Arahy.IZ5HKP*, *Arahy.Q05SK2*), several ferric reduction oxidase 2 proteins (*Arahy.SC8724*, *Arahy.1V3YN0*, *Arahy.SV1BLK*), and others such as protein kinase superfamily protein (Arahy.740L49), glycosyl group transferase (*Arahy.FMT16N*), and a U-box domain containing protein (*Arahy.D83LV0*). A fourth significant QTL (*p* < 8.37 × 10^−6^) was located on the chromosome B08 (AX-177643583) position located around 128130441 identified 13 candidate genes nearby, including a protein phosphatase 2A regulatory subunit B (*Arahy.77V5P5*, *Arahy.CQ5G6U*), RNA-dependent RNA polymerase 1 (*Arahy.MIQ9L3*), DEAD-box ATP-dependent RNA helicase-like protein (*Arahy.B0I1EQ*), transcription elongation factor S-II protein (*Arahy.8TMD5Z*), RING zinc finger protein (*Arahy.32TLHI*), CTC-interacting domain (*Arahy.7NC9IZ*), oxygen-evolving complex related protein (*Arahy.22L7FB*), cyclin a2 (*Arahy.N5YUQU*), and syntaxin-32 like protein (*Arahy.YQ853S*).

#### 3.3.2. Relative Water Content Stage 2

RWC stage 2 identified 31 suggestive SNPs on chromosomes A02 (6), A03 (3), A07 (3), A08 (1), A09 (3), B02 (7), B03 (1), B09 (6), B10 (1), and 2 significant SNPs. One significant SNP (*p* < 1.07 × 10^−6^) was located on chromosome A07 (AX-177638932) at position 66962106. Search for genes identified 10 candidate genes were located within proximity revealed including several myo-inositol oxygenase 2 and 4 (*Arahy.L7EMJP*, *Arahy.60DFDF*, *Arahy.TT4I2S*, *Arahy.30QA0S*), sucrose transporter 2 and 4 (*Arahy.QR9PB3*, *Arahy.5UJ4FC*), a MATE efflux family protein (*Arahy.2WQJ7Q*), a putative cyclin-D6-1 like protein (*Arahy.ZZZ0YY*), and several unknown proteins (*Arahy.3D8JQZ*, *Arahy.5B2J8N*). A second significant SNP (*p* < 6.75 × 10^−6^) on chromosome B03 (AX-147245281) at position around 120244146 identified 11 candidate genes, including a *myb*-transcription factor (*Arahy.0KDF2J*), signaling-related proteins such as calcium-dependent protein kinase 2 (*Arahy.H71TAS*), C3HC4-type RING zinc finger protein (*Arahy.9169VS*), tyrosyl-DNA phosphodiesterase-related protein (*Arahy.XWK03B*), and Sec14p-like phosphatidylinositol transfer family protein (*Arahy.M16IE8*).

#### 3.3.3. Specific Leaf Area Stage 1

SLA stage 1 identified 31 suggestive SNPs on chromosomes A08 (1), A10 (1), B08 (1), B10 (28), and one significant SNP (*p* < 4.09 × 10^−5^) was located on chromosome B10 (AX-177639510) at position 109491122. The search for genes nearby resulted in five candidate genes including DNA binding protein (Arahy.5Q0VV8), DNAJ homologue (*Arahy.X806TD*), uricase-2 isozyme (*Arahy.BN00BV*), iron-sulfur cluster assembly protein (*Arahy.P2449T*), and myosin XI (*Arahy.RG5XV3*). SLA stage 2 identified 26 suggestive SNPs on chromosomes A04 (3), A05 (1), A07 (3), B08 (2), B09 (17), and no significant SNP. LDMC stage 1 identified 28 suggestive SNPs on chromosomes A07 (2), B08 (24), B10 (2), and no significant QTL. Therefore, further analysis for LDMC stages 1 and 2 was not continued.

#### 3.3.4. Pod Count Number

Pod count identified four suggestive SNPs on chromosomes, namely A02 (1), A06 (2), B03 (1), and four significant SNPs. One significant SNP (*p* < 2.05 × 10^−6^) was located on chromosome A03 (AX-176823743) at position 112316851. The search for nearby genes identified five candidate genes including serine/threonine dehydratase serine racemase (*Arahy.HJ1545*), protein kinase (*Arahy.I232SR*), replication protein (*Arahy.D8EMDP*), and unknown proteins (*Arahy.QW3B2M*, *Arahy.X9VCK3*). Another significant SNP (*p* < 4.54 × 10^−5^) was located on chromosome A05 (AX-176803155) at position 35863460, with 11 candidate genes nearby, including receptor kinase 2 (*Arahy.AE9D0W*, *Arahy.XZQ3GI*, *Arahy.RK9A58*), pyruvate kinase (*Arahy.HI4HR4*), inositol-tetrakisphosphate kinase-like (*Arahy.V1YCRI*), phytosulfokine 4 precursor (*Arahy.KSYJ7I*), phosphotransferase (*Arahy.3J1H0B*), pyruvate kinase (*Arahy.HI4HR4*), and *myb*-like protein (*Arahy.2Q65GK*). A third significant SNP (*p* < 2.07 × 10^−5^) was located on chromosome B04 (AX-176821819) at position 33641851, and a search for nearby genes identified one candidate gene of unknown function (*Arahy.CPK3HX*). A fourth significant SNP (*p* < 1.10 × 10^−6^) was located on chromosome B05 (AX-176792912) at position 131612302. The search for nearby genes identified nine candidate genes including caffeic acid O-methyltransferase 1 (*Arahy.555E9X*), peptide/nitrate transporter (*Arahy.R2I5HX*), and cytochrome P450 (*Arahy.370AXC*), Got1/Sft2-like vesicle transport protein (*Arahy.U9G0PY*) and several uncharacterized proteins (*Arahy.B3S8NN*, *Arahy.KSCZ0Y*, *Arahy.CN16C1*).

#### 3.3.5. Dry Pod Weight Measurements

Pod dry weight identified two suggestive SNPs on A06 (1), B10 (1) and six significant SNPs. One significant SNP (*p* < 2.86 × 10^−5^) was on chromosome A05 (AX-176806959) at position 1298032. The search for nearby genes resulted in 20 candidate genes including PATATIN-like protein 5 (*Arahy.KDJI7F*, *Arahy.42C60E*, *Arahy.D6LT1N*), transcription factor MYB48 (*Arahy.J6YIRG*, *Arahy.M6TNBV*), heat shock transcription factor A2 (*Arahy.BB0AZK*, *Arahy.3757PS*), SNARE associated Golgi protein (*Arahy.J5SMTG*), ATP-binding ABC transporter (*Arahy.5666S0*), and inositol-tetrakisphosphate 1-kinase (*Arahy.BJ65FS*). A second significant SNP (*p* < 1.21 × 10^−6^) was on chromosome A07 (AX-176815659) at position 605413. The search for nearby genes located 41 candidate genes including oligopeptide transporter 1 and 5 (*Arahy.BL3QR7*, *Arahy.UU5TC6*, *Arahy.6WW0WD*, *Arahy.CZ34TI*), vacuolar protein-sorting protein (*Arahy.I2QCNX*, *Arahy.NWI6KJ*), disease-resistant proteins (*Arahy.K5EKT0*, *Arahy.M76BEQ*), chaperonin-like and heat shock protein (*Arahy.V44IJD*, *Arahy.B8WNJH*), ATP binding/protein serine/threonine kinase (*Arahy.VAAE0N*), ABC transporter (*Arahy.GGDJ84*), RING-H2 finger protein 2B (*Arahy.1WFS8V*), and LEA protein (*Arahy.0TKI11*). A third significant SNP (*p* < 4.92 × 10^−7^) was on chromosome A08 (AX-176792556) at position 1053912. Genes located nearby identified 15 candidate genes including BEL1-like homeodomain proteins (*Arahy.H9G0X7*, *Arahy.PU29YL*), transcription factor bHLH123-like (*Arahy.VRQ703*), BZIP transcription factor (*Arahy.VA7IIE*), protein kinase (*Arahy.X8RB79*), and cytochrome P450 (*Arahy.E2W35F*). A fourth significant SNP (*p* < 1.02 × 10^−5^) was on chromosome B06 (AX-176809834) at position 2362556. Genes located nearby revealed 24 candidate genes including F-box proteins (*Arahy.9KV828*, *Arahy.SSYA5T*, *Arahy.C9YQVF*), terpene synthases (*Arahy.PN0N9Z*, *Arahy.KA44CE*), PATATIN-like protein 9 (*Arahy.D7XM6E*), transcription factor TT8-like (*Arahy.4XX97Y*), glutathione S-transferase tau 5 (*Arahy.Z7I4MG*), chalcone-flavanone isomerase (*Arahy.EWUD7T*), and aluminum-activated malate transporter (*Arahy.YGE525*). A fifth (*p* < 9.83 × 10^−6^) was on chromosome B09 (AX-177642049) at position 120728154. The gene search found 26 candidate genes including ADP-ribosylation factor GTPase-activating protein (*Arahy.KN9S33*, *Arahy.PU4PH6*), malate dehydrogenase (*Arahy.WNG3H8*, *Arahy.GYV5V7*), galactosyltransferase (*Arahy.XNH8SK*), ubiquitin carboxyl-terminal hydrolase (*Arahy.RPY2B8*), actin depolymerizing factor 6 (*Arahy.YEWY3A*), methyl transferase (*Arahy.HTZ8JA*), and receptor-like protein kinase 2 (*Arahy.WU9FE7*). A sixth significant SNP (*p* < 2.42 × 10^−5^) was on chromosome B10 (AX-176819260) at position 66768283. The search for nearby genes identified 6 candidate genes including SufS subfamily cysteine desulfurase (*Arahy.K5CIRV*, *Arahy.RY2JMZ*), glyceraldehyde-3-phosphate dehydrogenase (*Arahy.6W2CKZ*), ubiquitin-protein ligase (*Arahy.JDS371*), and cyclin-dependent kinase inhibitor (*Arahy.DEJ7H2*).

#### 3.3.6. Determination of Drought Ratings

The drought rating identified 29 suggestive SNPs on chromosomes A01 (1), A04 (2), A05 (13), A06 (1), B04 (3), B05 (7), B06 (2), and 1 significant QTL (*p* < 4.83 × 10^−5^) on chromosome A06 with SNP identification (AX-147224451) at position 4994882. Gene search near proximity of marker revealed 31 candidate genes including protein AUXIN RESPONSE 4-like (*Arahy.ST8CS6*), chaperone DNAJ-domain (*Arahy.LJ09F6*), mannosyl glycoprotein endo-β-mannosidase (*Arahy.MMBS5J*), sucrose nonfermenting 4-like protein (*Arahy.B5S4LJ*), and others.

## 4. Discussion

The U.S. peanut mini-core collection represents a diverse genetic panel from six different botanical types, with sources from variable environments. Genome-wide association studies utilizing the U.S. peanut mini-core collection have been affective in identifying significant QTLs for specific traits in peanuts [10,11,12] due to the diversity of evaluation that has higher resolution and identifies more genomics regions than biparental RIL populations. The application of middle-season drought utilizing environmentally controlled rainout shelters provide a tight control over water availability and allow a linear plant response to a soil moisture dry-down over the period of several weeks that has the potential to significantly reduce yield. Water use efficiency (WUE) is an important trait in breeding for drought tolerance. RWC and SLA are surrogate traits for WUE that can reasonably be measured in field studies to identify drought-tolerant lines [25] and the evaluating leaf water status [26]. Changes in water leaf content as well as leaf morphology represent drought tolerance responses that can encompass a range of downstream biochemical and genetics responses [27].

In this study, we identified 126 suggestive SNPs and 18 significant SNPs with a total of 317 candidate genes for RWC (stages 1 and 2), SLA (stages 1 and 2), pod count, pod weight, and drought rating. SLA stages 1 and 2, and LDMC stage 2 have no significant correlations with pod weight, pod count, and drought rating. RWC (stages 1 and 2) were highly correlated to pod count, pod weight, and drought rating (0.23, 0.18, −0.33, and 0.26, 0.33, −0.41). RWC at 14 days after the initiation of progressive drought (stage 2), has been shown to be an important stage when the drought-tolerant peanut lines can be separated from susceptible lines [28]. Zhang et al. [29] showed that at 2 weeks after drought treatment, peanut responses have the highest relationship of physiological trait (such as photosynthesis) to yield, biomass, drought rating, and gas exchange, respectively (0.65, 0.65, −0.90, 0.69). Peanuts may respond in such a manner resulting in a mini drought recovery or an adjustment to the recognition of mild drought stress.

Search for candidate genes for RWC (stage 2) included myo-inositol oxygenase and its elevated gene expression improved drought tolerance in rice by scavenging reactive oxygen species [30]. In leaves, sucrose is an important photosynthetic product that requires sucrose transport to be distributed as a signaling molecule and for osmoregulation. Several sucrose transporters were identified near a significant SNP marker (AX-177638932) on chromosome A07. In rice, *OsSWEET13* and *OsSWEET15* sucrose transporters showed elevated gene expression under drought stress [31]. A multidrug and toxic compound extrusion (MATE) efflux family protein was identified near this SNP marker. MATE transporters are involved in development, stress response, leaf senescence, and various metabolite transport [32]. A putative cyclin-D6 protein was also identified. Cyclin-D6 is involved in cortex/endodermis asymmetric stem cell division in many plant tissues, including leaves. Its expression is tightly controlled by regulators for specific and temporal asymmetric cell division [33]. Genes surrounding significant SNP marker (AX-147245281) on chromosome B03 identified signaling proteins such as calcium-dependent protein kinase (CDPK or DPK) which are necessary to modulate and regulate target genes, transcription factors, ion channels, and enzymes to adapt to various abiotic stress conditions [34]. A C3HC4-type RING zinc finger protein was also identified. RING zinc finger proteins play criterial roles in plant growth and development and respond to various abiotic stresses, including drought [35]. A tyrosyl-DNA phosphodiesterase (Tdp) was also identified. Under drought and high temperature stress, plants accumulate a high number of non-specific covalent intermediates between topoisomerase I and DNA that are processed by Tdp1 [36]. A Sec14-like phosphatidylinositol transfer protein (PITP) was also identified. *Sec14-PITPs* can recognize, bind, and transfer small lipophilic molecules between membrane and non-vesical organelles [37,38].

In agronomic terms, yield is an important criterion of drought tolerance. The pod dry weight identified six significant SNP markers. For the SNP marker (AX-176806959) on chromosome A05, a PATATIN-like protein (PLP) gene was identified nearby which encodes a galactolipid acyl hydrolase (EC 3.1.1.26) and is differentially expressed under drought [39]. Genome sequence search in peanut identified 49 PLP family members with 26 in *A. hypogaea*, 11 in *A. duranensis*, and 12 in *A. ipaensis* with gene expression levels that are variable in different plant tissues [40]. Transcription factor *MYB48* was also identified as functions to regulate flavonol biosynthesis. The overexpression of maize *MYB48* in transgenic *Arabidopsis* plants conferred drought tolerance [41]. Evaluating the U.S. mini-core collection, a major seed size QTL on chromosome A05 was identified in a different study [8]. In this study, candidate genes were identified near another significant SNP (AX-176815659) on chromosome A07, which included oligopeptide transporters (OTPs). OTP family consists of a group of proton-coupled symporters and their roles include metal homeostasis, nitrogen level maintenance, and sulfur distribution [42]. Genome wide analysis identified 40 candidate *Ah* (*A. hypogaea*) *OTP* genes and a subset of these genes were induced by Fe deficiency [43]. In our study, another significant QTL (AX-176792556) was present on chromosome A08 with 15 candidate genes association. Examples include Inositol-pentakisphosphate 2-kinase, BEL1-like homeodomain protein, cytochrome P450, and UDP-glycosyltransferase. Significant QTL was discovered on chromosome B06 for pod weight [7]. In this study, a significant QTL (AX-176809834) for pod weight was identified on chromosome B06. The search for nearby genes identified 24 candidate genes, including several F-box proteins (*Arahy.9KV828*, *Arahy.SSYA5T*, *Arahy.C9YQVF*). An F-box protein in *Arabidopsis*, *MAX2*, regulates drought tolerance plant responses [44]. Terpene synthase (*Arahy.PN0N9Z*, *Arahy.KA44CE*) was also present, which has a primary role in the formation of low-molecular-weight terpene metabolites. The drought stress stimulates the production of terpenoid backbone and triterpenoids biosynthesis that promotes the synthesis of Saikosaponin in *Bupleurum chinense* roots [45]. Other genes within proximity included glutathione S-transferase, chalcone-flavanone isomerase, PATATIN-like protein, cyclin p1, transcription factor TT8-like, and aluminum-activated malate transporter. Significant QTLs were also identified on chromosomes B09 (AX-177642049) and B10 (AX-176819260), with 20 and 6 candidate genes, respectively.

In addition, the pod number count may represent the success of flower development and their pegging capacity to produce successful maturing pods. The conversion of flowers to mature pods is a major contributor to peanut high pod yield [46]. The ratio of flowers conversion to peg and peg to pod conversion are indicators of yield [47]. Drought during peanut pegging and pod filling stages can significantly reduce yield by reducing pod number rather than seed weight per pod [48]. A flush of flower production is often observed after the release of drought. QTL analysis identified four significant SNPs on chromosomes A03, A05, B04, and B05. A serine/threonine dehydratase or serine racemase was identified. In mammalian systems, D-serine intracellular levels are regulated by a serine racemate and have a high sequence homology with serine/threonine dehydratase [49]. Levels of D-serine have been shown to control the pollen tube growth in *Arabidopsis* [50]. On another significant SNP marker (AX-176803155), genes were identified such as pyruvate kinase (*PK*). An *OsPK2* encodes a plastic pyruvate kinase that is shown to be involved in rice endosperm starch biosynthesis as well as grain filling [51]. Another identified protein inositol-tetrakisphosphate kinase (*ITPK1*) was identified. In *Arabidopsis*, ITPK1 acts as a phosphotransferase that controls phosphate signaling [52]. A phytosulfokine (*PSK*) precursor was identified. In *Arabidopsis*, the PSK precursor processing by subtilase *SBT3.8* combined with PSK signaling improved drought stress [53]. Near significant SNP marker (AX-176792912) on chromosome B05, a caffeic acid O-methyltransferase (COMT) candidate gene was identified. COMT is one of the major enzymes involved in lignin biosynthesis and 20 out of 33 analyzed showed differential gene expression under various types of biotic stress in rice [54].

Drought rating was performed after 4 weeks of progressive drought and at the hottest time of the day (2–4 pm) to observe fine phenotypic differences among peanut genotypes. Leaf wilting observation is easily visible, representing the response of the peanut that loses the ability to replenish water lost through transpiration. Peanut leaves tend to either fold, roll, or droop down [55]. A significant QTL was identified on chromosome B06 (AX-147224451) with 31 candidate genes identified. These included Chaperone DnaJ-domain protein, seryl-tRNA synthetase, AUXIN RESPONSE 4-like, sucrose nonfermenting 4-like protein, and prefoldin.

## 5. Conclusions

The application of middle-season drought utilizing environmentally controlled rainout shelters has been effective to correlate yield quality traits. Evaluating the U.S. peanut mini-core collection for drought tolerance enables the discovery of significant QTLs and candidate genes. In this study, six significant QTLs were identified for pod weight of which 1 was identified on B06 in other studies. Pod count identified four significant QTLs, two of which were present in LGs A05 and B05. RWC stages 1 and 2 were directly correlated with pod weight and pod count and negatively correlated with drought rate. A total of 18 significant QTLs were identified for pod weight, pod count, RWC, SLA, and drought rating and 317 candidate genes. Many QTLs were novel and one QTL on B06 for pod weight was observed in other studies. These newly identified genomic regions and candidate genes provide breeding targets for applications in molecular marker-assisted peanut breeding. Further research will elucidate gene functions to facilitate the development of drought-tolerant peanut varieties.

## Figures and Tables

**Table 1:** Variance components and broad-sense heritability estimates for 9 measured traits in 124 genotypes mainly selected from the U.S. peanut mini-core collection.

Traits ^1^	Dry Wt	Pod #	Rate	RWC1	SLA1	LDMC1	RWC2	SLA2	LDMC2
*σ* ^2^ *_e_*	2446.48	2101.65	0.07	3.99	84.14	0	0.67	174.55	0.00621
*σ* ^2^ *_g_*	2831.4	2059.81	0.15	0.46	68.56	0.00009	2.54	37.06	0
*σ* ^2^ *_g×e_*	172.19	84.02	0.16	3.66	108.75	0.00016	4.94	33.79	0.0008
*σ* ^2^ *_error_*	4566.38	4018.68	1.21	11.41	573.05	0.00057	20.79	616.79	0.00056
Heritability	69.75	66.31	28.89	14.11	40.17	41.18	37.71	35.18	0

^1^ Dry Wt: pod weight (grams) after curing; Pod #: pod count; Rate: drought rating of peanut plants at the peak of stress; RWC1 or RWC2: relative water content at stages 1 or 2; SLA1 or SLA2: specific leaf area at stages 1 or 2; LDMC1 or LDMC2: leaf dry matter content at stages 1 or 2.

## Data Availability

Data are contained within the article and Supplementary Material.

## Supplementary Materials

The following supporting information can be downloaded at: https://www.mdpi.com/article/10.3390/genes15070868/s1, Table S1: Information of the diversity panel consisting of 124 accessions used in this GWAS study; Figure S1: Correlation matrix for yield-related traits for 2017 and 2018; Table S2: Significant SNPs for physiological and yield-related traits; Table S3: Co-localize genomic regions and associated genes for physiology and yield-related traits discovered in this GWAS study.
