# Supplementary material for: Genome-Wide Association Analysis Identified Quantitative Trait Loci (QTLs) Underlying Drought-Related Traits in Cultivated Peanut (Arachis hypogaea L.)
Source: Genes (Basel). 2024 Jul 2;15(7):868. doi: 10.3390/genes15070868 (PMC11276114; doi:10.3390/genes15070868)
Supplement: Supplementary file 1 [file genes-15-00868-s001.zip › Supplementary Fig 1 Correlation 2017 & 2018.pdf]

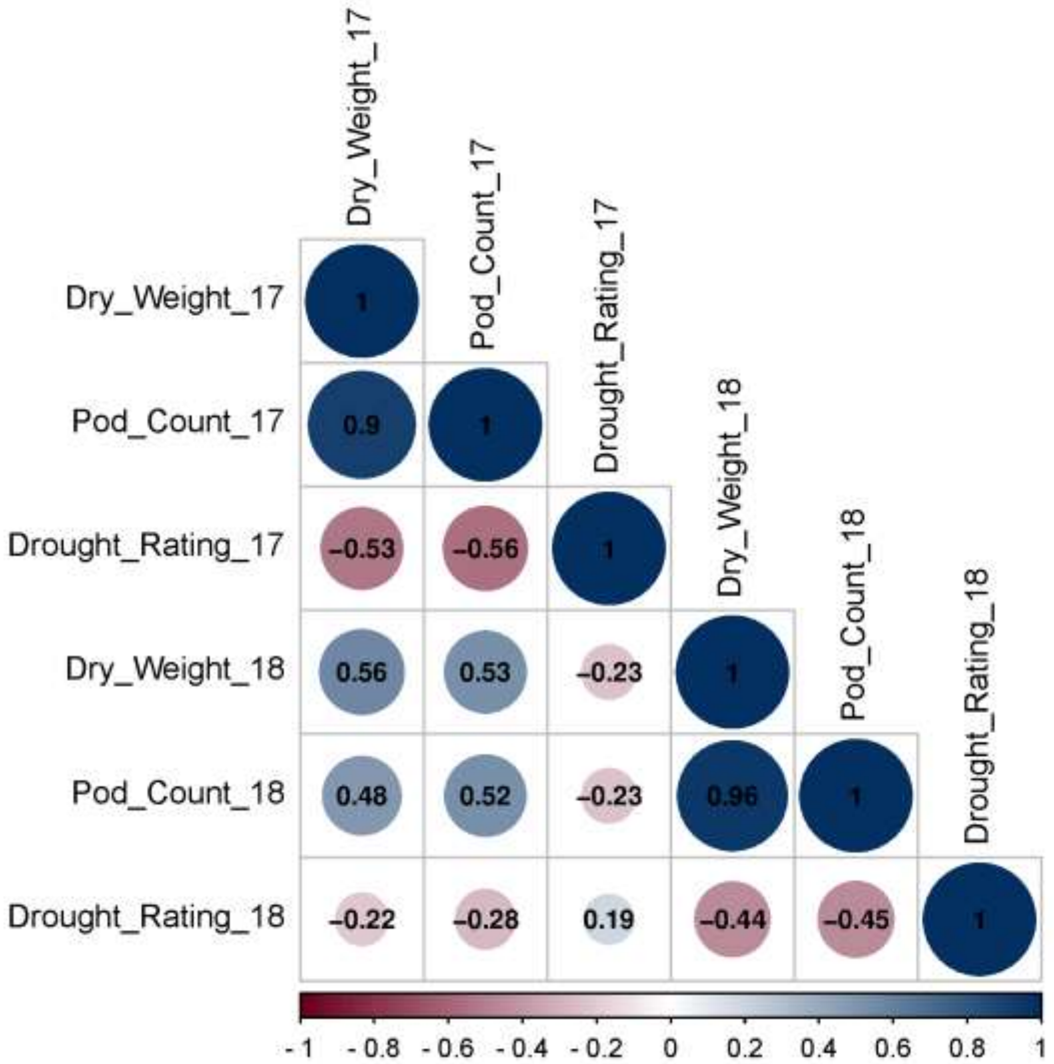

Supplementary Fig. 1 Correlation of yield related straits, dry pod weight and pod count, along with drought rating for 2017 and 2018. Pod count and dry pod weight for 2018 are approximately half (0.56 and 0.53) of 2017 pod count and pod dry. Drought rating is negatively correlated with both dry pod weight and pod count at -0.22 and -0.28 for 2018, approximately half reduced from 2017 (-0.53 and -0.56). The size and number of the circle indicate the strength of the relationship and correlation coefficient (r). Blue color indicates a positive relationship and red indicates a negative relationship between traits.
